# Editorial: Type III interferons: Emerging roles beyond antiviral barrier defense

**DOI:** 10.3389/fimmu.2022.1030812

**Published:** 2022-09-14

**Authors:** Steeve Boulant, Adriana Forero, Deanna M. Santer, Achille Broggi

**Affiliations:** ^1^ Department of Molecular Genetics & Microbiology University of Florida, College of Medicine, Gainesville, FL, United States; ^2^ Department of Microbial Infection and Immunity, College of Medicine, The Ohio State University, Columbus, OH, United States; ^3^ Infectious Diseases Institute, The Ohio State University, Columbus, OH, United States; ^4^ The James Comprehensive Cancer Center, The Ohio State University, Columbus, OH, United States; ^5^ Department of Immunology, University of Manitoba, Winnipeg, MB, Canada; ^6^ Aix Marseille Université, CNRS, INSERM, Centre d’Immunologie de Marseille-Luminy (CIML), Marseille, France

**Keywords:** interferon, IFN-lambda, antiviral, IBD, infection, mucosae, IFN-λ4, vaccine

## Introduction

Type III interferons or IFN-λs, are composed of 4 members: IFN-λ1, IFN-λ2, IFN-λ3, and IFN-λ4. IFN-λs are master protectors against mucosal viral infections due to the preferential expression of their cognate receptor, IFNLR1, to cells of epithelial lineage and to a restricted pool of immune cells. IFN-λ receptor activation induces intracellular signaling that overlaps with that of type I interferons (IFN-I) and culminates with the induction of Interferon-Stimulated-Genes (ISG) with antiviral functions. However, several studies have uncovered non-redundant functions of IFN-λs, compared to IFN-I, that go beyond viral restriction at epithelial barriers. In this topic, we highlighted several emerging aspects of IFN-λ biology, including: their ability to restrict non-viral infections, the mechanisms by which they modulate immune responses, and the enigmatic role of human IFN-λ4.

## Immunomodulatory roles of IFN-λs

One of the defining characteristics of IFN-λs is their ability to induce a targeted response in the barrier epithelia without stimulating excessive immune cell recruitment which can lead to immunopathology. This property of IFN-λs makes them central regulators of inflammation during viral and non-viral diseases. On the one hand, in addition to inducing antiviral gene programs, IFN-λs can dampen immune cell-mediated inflammation and promote physiological intestinal epithelial cell functions. On the other hand, IFN-λs can fuel the immunopathology of inflammatory diseases by preventing cell differentiation and proliferation and thus dampening barrier repair. In this Research Topic, Wallace et al. describe how regional identity and timing of disease progression contribute to the outcomes of exposure to IFN-λs in the gastrointestinal tract. They summarize how dual regulation of epithelial and immune cells contributes to protective and pathogenic outcomes during inflammatory bowel diseases (IBD). IFN-λs can also modulate the activity of several immune cells and influence the pathogenesis of autoimmune diseases as comprehensively described by Manivasagam and Klein. Mouse models provided a robust genetic system to study the distribution of *Ifnlr1* across immune cells. In humans, however, our understanding of the cells contributing to the production of IFN-λs and the capacity of immune cells to respond to IFN-λs is limited. Mallampalli et al. demonstrate that human macrophages are an important source of IFN-λs following Influenza A virus (IAV) infection. They also show that human alveolar macrophages and monocyte-derived macrophages express functional levels of *IFNLR1* and can thus mount protective responses against IAV infection in response to IFN-λs.

The observation that IFN-λs modulate host inflammation opens new questions regarding their therapeutic applications in infectious diseases. In their original paper, Ye et al. build upon their previous work that demonstrates a cell-extrinsic role for IFN-λs in boosting adaptive immune responses to IAV. IFN-λs stimulate thymic stromal lymphopoietin (TSLP) release by specialized respiratory epithelial cells to activate dendritic cells and enhance adaptive immune responses following vaccination. Here, they demonstrate that this adjuvant effect is restricted to the mucosa as other non-mucosal routes of vaccination are not affected by IFN-λs, and that the IFN-λs/TSLP axis governs vaccine-induced immunity following intra-rectal vaccination, expanding upon the therapeutic potential of IFN-λs for the management of other mucosal pathogens.

Overall IFN-λs’ ability to shape immunity is increasingly appreciated. Several contributions to this topic highlight the emerging role of IFN-λs as immune regulators and raise interesting questions, such as what are the differences between the mouse and human IFN-λs biology, and what other indirect connections between IFN-λs and immune responses exist.

## The enigmatic role of IFN-λ4 in humans

IFN-λ4 was the latest addition to the IFN-λs family and its functions have been a matter of debate since its discovery. Although the *IFNL4* gene is present in most higher mammals, excluding mice, in humans, only a subset of individuals is genetically able to produce IFN-λ4. Single nucleotide polymorphisms in the *IFNL4* locus impede IFN-λ4 translation by causing a frameshift in the first exon. However, a significant proportion of humans, harbor functional *IFNL4* alleles. The heterogeneity in IFN-λ4 expression is clinically relevant as IFN-λ4 expression predicts host susceptibility to chronic Hepatitis C virus (HCV) infection and decreased clearance of HCV. This characteristic of IFN-λ4 is paradoxical: IFN-λ4 stimulates ISG transcription to induce an antiviral state in target cells, yet it prevents the clearance of a chronic virus infection. Thus, understanding the biological processes uniquely affected by IFN-λ4 is of utmost interest. In this Research Topic, Guo et al. compare the antiviral functions of distinct IFN-λ4 variants by following the kinetics of IFN-λ-receptor signaling, downstream ISG expression, and the ability to establish an antiviral state. By comparing human IFN-λ4 variants with their non-human primate counterparts, the authors reveal that IFN-λ4s are less potent than the other IFN-λs and display a wide range of activities, implicating that the expression of different variants can influence the susceptibility to infectious diseases.

However, how IFN-λ4 drives detrimental responses during infection is still unknown. One step toward the answer comes from Onabajo et al. who analyze the behavior of hepatocytes expressing IFN-λ4 and describe that IFN-λ4 is a misfolded protein, whose expression induces ER stress responses associated with cell cycle arrest, apoptosis, and decreased cell proliferation, potentially explaining the pro-HCV effects of IFN-λ4. Overall, the clinical relevance of IFN-λ4 is far from elucidated. This topic has contributed additional insights which point to a model in which a combination between ER stress responses and IFN responses may be at the base of IFN-λ4 detrimental roles.

## IFN-λs and non-viral infectious pathogens

Interferons have been classically viewed as potent antiviral cytokines. The induction of ISG inhibits the replication of viral pathogens by modifying intracellular biological processes necessary for pathogen dissemination. More recent studies have demonstrated that IFNs can also modify how cells respond to intracellular bacterial infections. In this Research Topic, Alphonse et al. highlight our understanding of the central role that IFN-λs play in the control of bacterial pathogens. Their work summarizes the mechanisms by which bacterial recognition drives IFN-λs production, how IFN-λs shape the cell-intrinsic control of bacterial replication, and the ability of IFN-λs to shape the inflammatory response to bacterial challenges. Further work is needed to expand our understanding of IFN-λ and bacterial interactions.

## Conclusion

IFN-λs were historically believed to be a redundant addition to the interferon arsenal present in epithelial cells. Their originality resided in two critical differences 1) their receptor is specific to mucosal surfaces, and 2) they confer anti-viral activity with different kinetics compared to type I IFNs. We have now started to peel back the layers and reveal unique IFN-λs-specific functions that might be even more important than their primary anti-viral function. We now know that IFN-λs are excellent immune modulators involved in regulating the response to infectious agents, autoimmunity, and chronic inflammatory disorders. Finally, the biology of the shyest IFN-λ, IFN-λ4, is starting to be unraveled. However, our understanding of IFN-λ4 biology is in its infancy.

IFN-λs need to be studied beyond their canonical anti-viral functions as several pieces of evidence suggest that these cytokines have context-specific functions and act differently in different cell types. It is critical to understand how IFN-λs act in individual cell types and to integrate this understanding to define the role of IFN-λs at the tissue level as a mediator of intrinsic local protection and coordinator of tissue-specific immune cells.

## Author contributions

All authors listed have made a substantial, direct, and intellectual contribution to the work and approved it for publication.

## Funding

AB is supported by the excellence initiative of Aix Marseille Universit.-A*Midex, a French “investissements d’Avenir” program: AMX-20-CE-01; the FRM amor.age de jeunes equipes grant AJE202010012468; and the ANR-JCJC grant “INTERMICI” ANR-21-CE15-0022. This work was supported by research grant starting package from the University of Florida, College of Medicine, to SB DMS was supported by the University of Manitoba, Natural Sciences and Engineering Research Council of Canada, Research Manitoba, Children's Hospital Research Institute of Manitoba and Canadian Institutes of Health Research (GA1- 177700). AF was supported by OSU funds for Advancing Research in Infection and Immunity.

## Conflict of interest

The authors declare that the research was conducted in the absence of any commercial or financial relationships that could be construed as a potential conflict of interest.

## Publisher’s note

All claims expressed in this article are solely those of the authors and do not necessarily represent those of their affiliated organizations, or those of the publisher, the editors and the reviewers. Any product that may be evaluated in this article, or claim that may be made by its manufacturer, is not guaranteed or endorsed by the publisher.

